# Supplementary material for: Functional Exploration of African Colorectal Cancer Patients Using Personalised Drosophila Avatars
Source: bioRxiv. 2026 Mar 30:2026.03.26.714433. Preprint. [Version 1] doi: 10.64898/2026.03.26.714433 (PMC13060073; doi:10.64898/2026.03.26.714433)
Supplement: Supplement 1 [file NIHPP2026.03.26.714433v1-supplement-1.pdf]

bioRxiv preprint doi: https://doi.org/10.64898/2026.03.26.714433; this version posted March 30, 2026. The copyright holder for this preprint (which was not certified by peer review) is the author/funder, who has granted bioRxiv a license to display the preprint in perpetuity. It is made available under aCC-BY 4.0 International license.

Supplemental Table 1- Reagents and tools

Reagent/Resource	Reference or Source	Identifier or Catalogue Number
Experimental Models		
RAP-N3 w⁻; RasV12, Egfr (P53, apc, Sox100B, ago, Tet, Daxx, mTor) ^{RNAi}/CTR	Cagan's lab	N/A
RAP-N4 w⁻; RasV12 (P53, apc, Sox100B, ago, osa, Lar, polo, Bnl) ^{RNAi}	Cagan's lab	N/A
RAP-N11 w⁻; RasV12, Egfr, Pi3K92E (P53, apc, pan) ^{RNAi}/CTR	Cagan's lab	N/A
w^{1118}	Cagan's lab	N/A
w⁻;; *byn*-GAL4, pTub-GAL80TS, UAS-GFP/TM6b GAL80	Cagan's lab	N/A
Drosophila Culture Reagent		
Methyl paraben	VWR Chemical	25604.290
D-glucose	VWR Chemicals	101174Y
Corn meal	Bodija (local market)	N/A
Soya bean	Bodija (local market)	N/A
Wheat	Bodija (local market)	N/A
Baker's yeast	STK ROYAL	LD5068H
Propionic acid	Guangdong Guanghua Sci-tech Co. ltd	20150331
Agar powder	Chaitanya Agro biotech PVT. LTD	RDM-1B/25-AA-01
Recombinant DNA		
Antibodies		
Phospho-p44/42 MAPK (Erk1/2) (Thr202/Tyr204)	Cell signalling Technology	9101S
Alexa Flour 555 goat anti-rabbit IgG (H + L)	Invitrogen	A-21428

bioRxiv preprint doi: https://doi.org/10.64898/2026.03.26.714433; this version posted March 30, 2026. The copyright holder for this preprint (which was not certified by peer review) is the author/funder, who has granted bioRxiv a license to display the preprint in perpetuity. It is made available under aCC-BY 4.0 International license.

Oligonucleotides and other sequence-based reagents		
Trx-2 PCR primer<br>Forward:<br>GTCTCCACATCTCCCATCCA<br>Reverse:<br>TTGACGCCGTTCTTGAGGAA	Inqaba Biotech WA	
Chemicals, Enzymes and other reagents		
Bovine serum albumin	Molychem	3120
Trametinib	Selleck chemicals	S2673
Regorafenib	Selleck chemicals	S1178
Paraformaldehyde	LOBA Chemie	0512800500
Hoechst/DAPI	Thermo Scientific	62249
Dimethyl sulfoxide (DMSO)	Molychem	14187
5,5′-dithiobis-2-nitro-benzoic acid (DTNB)	Ak Scientific	LC36490
Trizol	ZYMO Research	R2050-1-200
Folin-Ciocalteu’s phenol reagent	LOBA Chemie	0387000
Sodium hydroxide	LOBA Chemie	0589800
Sodium carbonate monohydrate	VWR chemicals	36485-36
Potassium sodium tartrate tetrahydrate	KLINCENT Laboratories LTD	6381-59-5
Copper sulphate	Guangdong Guanghua Sci-tech Co. Ltd	20190216
MTT (3-(4,5-dimethylthiazol-2-yl)-2,5-diphenyltetrazolium bromide	Ak Scientific	LC44417
2',7'-Dichlorodihydrofluorescein diacetate (DCFH-DA)	Santa Cruz Technology Inc.	Sc-209391
Trichloroacetic acid (TCA)	Molychem	19610
Dipotassium hydrogen phosphate (K_2HPO_4)	Guangdong Guanghua Sci-tech Co., Ltd.	1.01938.010
Potassium dihydrogen phosphate (KH_2PO_4)	Guangdong Guanghua Sci-tech Co. ltd	1.01863.020
L-glutathione reduced	Ak Scientific	TC31210

bioRxiv preprint doi: https://doi.org/10.64898/2026.03.26.714433; this version posted March 30, 2026. The copyright holder for this preprint (which was not certified by peer review) is the author/funder, who has granted bioRxiv a license to display the preprint in perpetuity. It is made available under aCC-BY 4.0 International license.

Sulfanilamide	Sigma Aldrich	1002867308 (S9251-100G)
Sucrose	VWR chemicals	27480
Sodium Chloride	MRS scientific	CO/31134
Triton X-100	Neutronco	4.0350
N-(1-naphthyl) ethylenediamine dihydrochloride	Ak Scientific	LC57989
Ethanol absolute	VWR chemicals	20821.321
Isopropanol	Sure chemical product LTD	P8104
Chloroform	Sure chemical product LTD	C4914
cDNA super mix kit	New England BioLab	E3010
qPCR Master mix	New England BioLab	M3003
Software		
Image J 1.54p (Fiji)		N/A
Graph pad prism v 9.0		N/A
Inkscape v 1.4.2		N/A
Image view v x64,3.7.6701		N/A
Power BI Desktop, Version 2.152.1057.0 64-bit (March 2026).	Microsoft	
Other		
Microscope digital camera SCMOS 05000KPA		Serial number: 16051000089
QuantStudio 5 Real-Time PCR System	Thermo Fisher Scientific	A34322
Zeiss Axioskop Fluorescent microscope	Zeiss	
Eppendorf centrifuge 5804 R	Eppendorf ®	
Spectramax plus 380 Microplate reader	Molecular devices	S/N 02728
Stereo microscope	StereoBlue-Euromex	N/A

bioRxiv preprint doi: https://doi.org/10.64898/2026.03.26.714433; this version posted March 30, 2026. The copyright holder for this preprint (which was not certified by peer review) is the author/funder, who has granted bioRxiv a license to display the preprint in perpetuity. It is made available under aCC-BY 4.0 International license.

Supplementary Information

Table S1: Sequences of single 21-nucleotide siRNA for knockdown of tumour suppressors.

Patient specific Avatar	Fly gene	# of transcripts	Antisense Sequence	DSIR Corrected Score
RAP-N3	*Sox100B*	1	TCATTCGAAGCTTCTCGGCGA	84.7
	Ago	3	TACAATGTCCACTGCGTCCTC	84.9
	Tet	6	TATCCATTTGCCGACCGTCGT	85.3
	Daxx	2	ACTATGACCGGAGTCTCCCTT	84.7
	mTor	2	TCTTCTGGACGTCTTCCGGCA	84.6
RAP-N4	*osa*	6	AGTGTATGCAAGTCTTGTCTG	85.1
	Lar	7	TACCTGACTATGTCTGAAGCT	84.5
	Sox100B	1	ATAAGTTTAGGAATACCGATG	84.7
	polo	2	TAGCGGCATTCGAACTCCGTA	84.5
	Bnl	3	TAAACAGATTACTATTGTTCG	84.8
RAP-N11	*Pan*	9	TCGTGCTAACTACTGGTGGAC	63.9

bioRxiv preprint doi: https://doi.org/10.64898/2026.03.26.714433; this version posted March 30, 2026. The copyright holder for this preprint (which was not certified by peer review) is the author/funder, who has granted bioRxiv a license to display the preprint in perpetuity. It is made available under aCC-BY 4.0 International license.

Table S2: Selected Nigerian patient-specific colorectal cancer driver genes, their *Drosophila* orthologues, and associated signalling pathways

Gene	*Drosophila* othologue	Transgenic construct	Genetic alterations N3	Genetic alterations N4	Genetic alterations N11	Signalling Pathway
KRAS	rasG12V	UAS-Transgene	Missense Mutation (putative driver)	Missense Mutation (putative driver)	Missense Mutation (putative driver)	RAS/MAPK Signalling
EGFR	ERBB2	UAS-Transgene	Missense Mutation (putative driver)	No alterations	Amplification	
PIK3CA	Pi3K92E	UAS-Transgene	No alterations	No alterations	Missense Mutation (putative driver)	PI3K/AKT/mTOR Pathway
MTOR	mTor	UAS-RNAi	Missense Mutation (unknown significance)	No alterations	No alterations	
FBXW7	ago	UAS-RNAi	No alterations	No alterations	Truncating Mutation (putative driver)	Notch Pathway
APC	Apc	UAS-RNAi	Truncating Mutation (putative driver)	Truncating Mutation (putative driver)	Truncating Mutation (putative driver)	Wnt Signalling
TCF7L2	pan	UAS-RNAi	No alterations	No alterations	Missense Mutation (unknown significance)	
TP53	p53	UAS-RNAi	Truncating Mutation (putative driver)	Missense Mutation (putative driver)	Truncating Mutation (putative driver)	Genome Integrity Pathway
SOX9	Sox100B	UAS-RNAi	Truncating Mutation (putative driver)	No alterations	Truncating Mutation (putative driver)	Other pathways
TET2	tet	UAS-RNAi	No alterations	No alterations	Inframe Mutation (unknown significance)	
DAXX	Daxx	UAS-RNAi	No alterations	No alterations	Truncating Mutation (putative driver)	
ARID1A	osa	UAS-RNAi	Truncating Mutation (putative driver)	No alterations	No alterations	
PLK2	polo	UAS-RNAi	Missense Mutation (unknown significance)	No alterations	No alterations	
PTPRT	lar	UAS-RNAi	Missense Mutation (putative driver)	No alterations	No alterations	
FGF3	Bnl	UAS-RNAi	Missense Mutation (unknown significance)	No alterations	No alterations	

Genetic Alteration

- Missense Mutation (putative driver)
- Amplification
- Truncating Mutation (putative driver)
- Inframe Mutation (unknown significance)
- No alterations
- Missense Mutation (unknown significance)

bioRxiv preprint doi: https://doi.org/10.64898/2026.03.26.714433; this version posted March 30, 2026. The copyright holder for this preprint (which was not certified by peer review) is the author/funder, who has granted bioRxiv a license to display the preprint in perpetuity. It is made available under aCC-BY 4.0 International license.

Table S3: Summary of the selected three patient-derived colorectal cancer Fly avatars and their genetic alterations

Avatar ID	Patient ID	Base	No. of mutations	shRNAs	Oncogenes
N3	pkc_crc_083	RAP	9	*Sox100B* (SOX9), *ago* (FBXW7), *Tet* (TET2), *Daxx* (DAXX), *mTor* (MTOR)	*Egfr* (ERBB2)
N4	pkc_crc_137	RAP	8	*osa* (ARID1A), *Sox* 100B (SOX9), *Lar* (PTPRT), *polo* (PLK2), *Bnl* (FGF3)	
N11	pkc_crc_079	RAP	6	*pan* (TCF7L2)	*Pi3K92E* (PIK3CA), *Egfr* (ERBB2)

bioRxiv preprint doi: https://doi.org/10.64898/2026.03.26.714433; this version posted March 30, 2026. The copyright holder for this preprint (which was not certified by peer review) is the author/funder, who has granted bioRxiv a license to display the preprint in perpetuity. It is made available under aCC-BY 4.0 International license.

Figure S1: Cloning vector for transgenesis

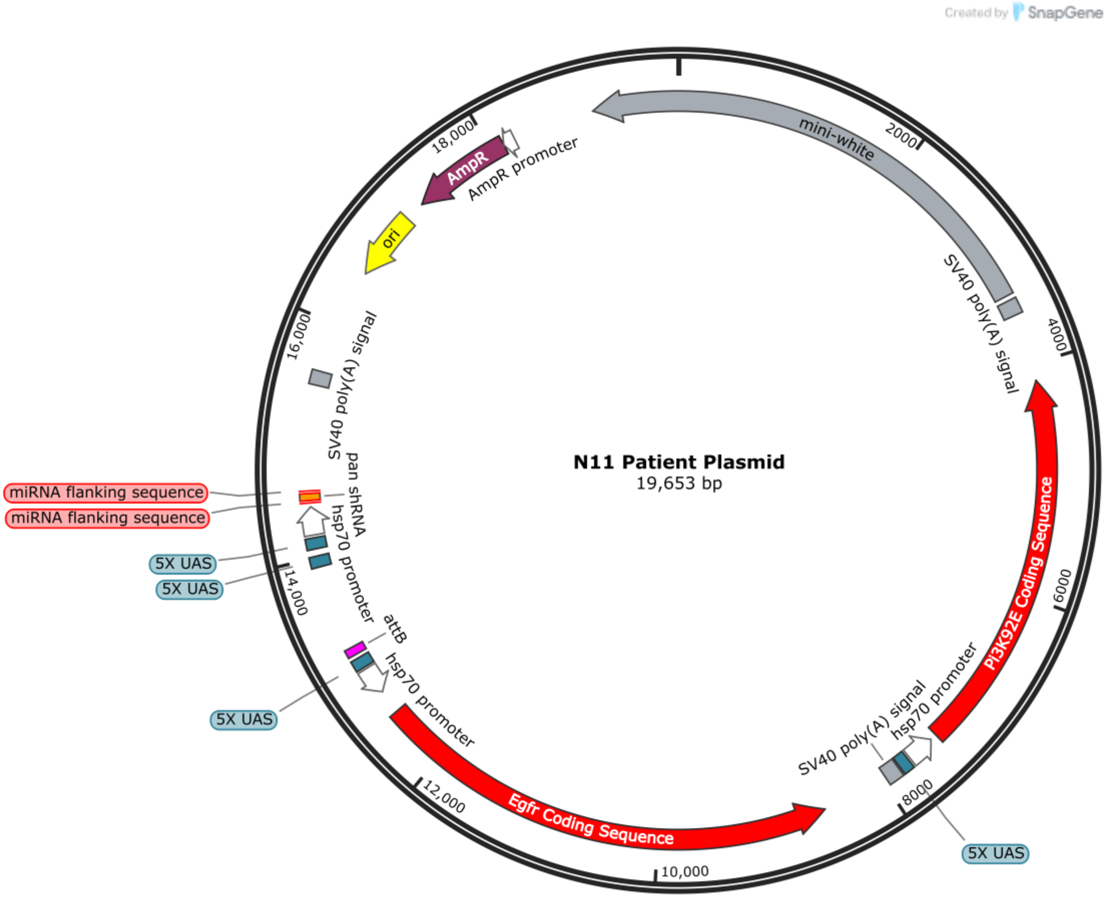

bioRxiv preprint doi: https://doi.org/10.64898/2026.03.26.714433; this version posted March 30, 2026. The copyright holder for this preprint (which was not certified by peer review) is the author/funder, who has granted bioRxiv a license to display the preprint in perpetuity. It is made available under aCC-BY 4.0 International license.

Figure S2: Spatial Intensity profile of phospho-ERK in regorafenib and trametinib fed *byn > RAP-N3*

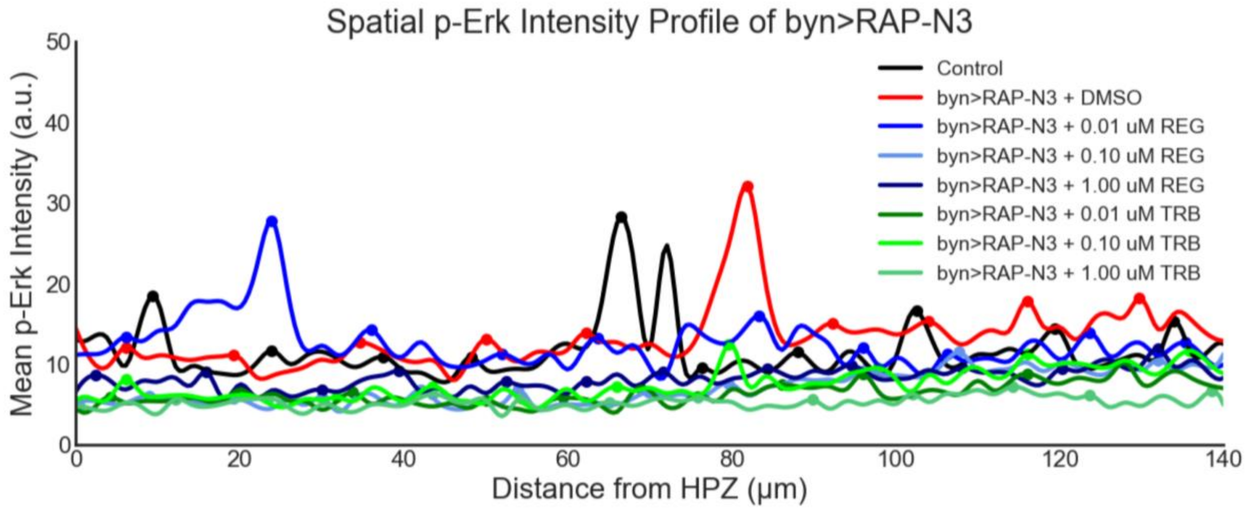

Figure S3: Spatial Intensity profile of phospho-ERK in regorafenib and trametinib fed *byn > RAP-N4*

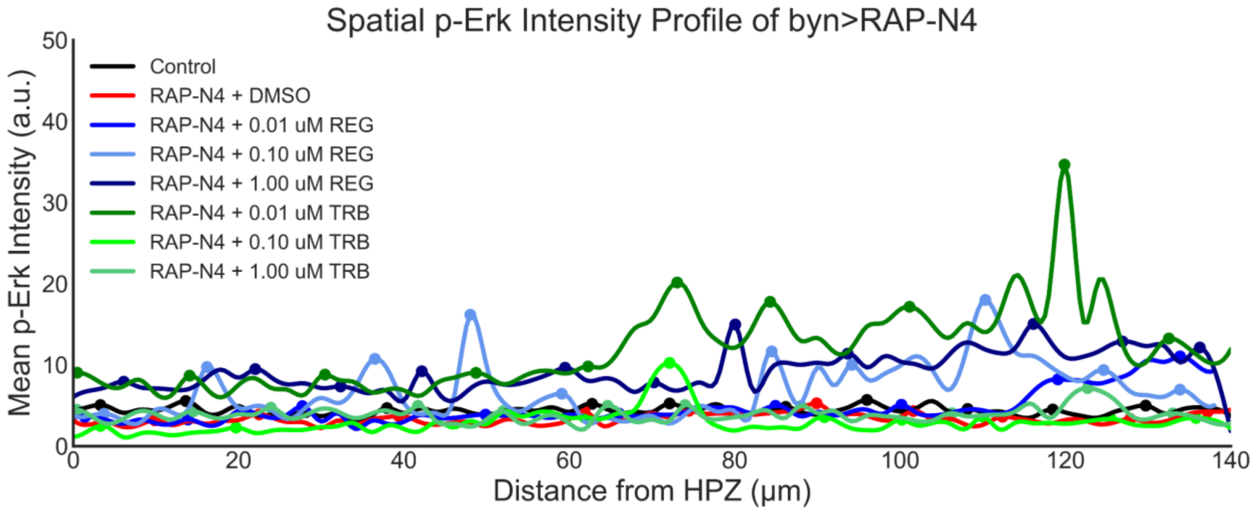

bioRxiv preprint doi: https://doi.org/10.64898/2026.03.26.714433; this version posted March 30, 2026. The copyright holder for this preprint (which was not certified by peer review) is the author/funder, who has granted bioRxiv a license to display the preprint in perpetuity. It is made available under aCC-BY 4.0 International license.

Figure S4: Spatial Intensity profiles of phospho-ERK in regorafenib and trametinib fed *byn > RAP-N11*

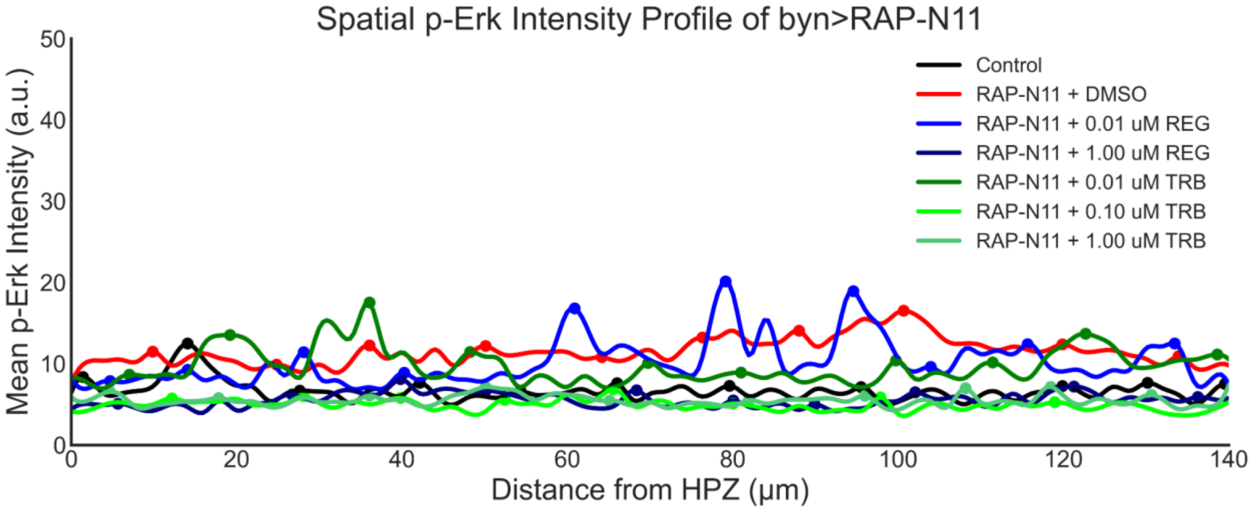

Note S1

Fly crosses

Crosses were done using virgin females of *byn-Gal4*, *Gal80(ts)*, *GFP*/*TM6b*, and male Transgene lines in the ratio 2:1. Flies were pre-mated for 48 hours at 27 ℃ before the various groupings. The experiment was carried out at 27 ℃, and the adult flies were moved out of the drug-containing diet after 24 hours of egg laying. Third instar larvae were collected for further studies.

Note S2

Temperature calibration

To determine the most appropriate condition for the fly model under study, temperature calibration of the Nigerian CRC avatar lines was carried out in different temperature conditions. Flies were pre-mated for 48 hours at 27℃ before the various groupings.

Four different conditions for calibration.

(1) The flies were left to mate for 24 hours at 27 ˚C, after which the embryo-containing diet was left at 27 °C until the third instar larva emerged.

(2) The flies were allowed to lay for 24 hours at 18 ˚C, then the embryo-containing diet was moved to 27 °C until the third instar larva emerged.

(3) The flies were allowed to lay for 24 hours at 18 ˚C. The embryo-containing diet was left at 18 °C for an additional 24 hours and then transferred to 27 °C until the third-instar larvae emerged.

(4) The flies were allowed to lay for 24 hours at 18 ˚C. The embryo-containing diet was left at 18 °C for an additional 48 hours and then moved to 27 °C until the third instar larva emerge.

Correlation Analysis for Relative gut size vs other biomarkers

Table S4: Correlation analysis of gut size and assayed biomarkers of regorafenib-treated *byn>RAP-N3* fly avatar.

	Relative Gut Size vs. ECLOSION	Relative Gut Size vs. TSH	Relative Gut Size vs. NPSH	Relative Gut Size vs. NO	Relative Gut Size vs. RONS	Relative Gut Size vs. MTT	Relative Gut Size vs. GFP Intensity
Pearson r							
r	-0.9910	0.1832	-0.2116	0.2791	0.1356	0.02199	-0.8312
95% confidence interval	-0.9998 to -0.6279	-0.9441 to 0.9730	-0.9745 to 0.9408	-0.9320 to 0.9779	-0.9492 to 0.9702	-0.9594 to 0.9627	-0.9963 to 0.6458
R squared	0.9820	0.03357	0.04475	0.07788	0.01839	0.0004835	0.6908
P value							
P (two-tailed)	0.009	0.82	0.79	0.72	0.86	0.98	0.17
P-value summary	**	ns	ns	ns	ns	ns	ns
Significant? (alpha = 0.05)	Yes	No	No	No	No	No	No

Table S5: Correlation analysis of gut size and assayed biomarkers of trametinib-treated *byn>RAP-N3* fly avatar.

	Relative Gut Size vs. ECLOSION	Relative Gut Size vs. TSH	Relative Gut Size vs. NPSH	Relative Gut Size vs. NO	Relative Gut Size vs. RONS	Relative Gut Size vs. MTT	Relative Gut Size vs. GFP Intensity
Pearson r							
r	-0.9562	0.5531	-0.1740	0.2269	0.07643	-0.5157	-0.9169
95% confidence interval	-0.9991 to 0.06051	-0.8710 to 0.9886	-0.9725 to 0.9451	-0.9389 to 0.9753	-0.9548 to 0.9665	-0.9874 to 0.8831	-0.9983 to 0.3722
R squared	0.9143	0.3059	0.03028	0.05148	0.005842	0.2659	0.8406
P value							
P (two-tailed)	0.04	0.45	0.83	0.77	0.92	0.48	0.08
P value summary	*	ns	ns	ns	ns	ns	ns
Significant? (alpha = 0.05)	Yes	No	No	No	No	No	No

Table S6: Correlation analysis of gut size and assayed biomarkers of regorafenib-treated *byn>RAP-N4* fly avatar.

	Relative Gut Size vs. ECLOSION	Relative Gut Size vs. TSH	Relative Gut Size vs. NPSH	Relative Gut Size vs. NO	Relative Gut Size vs. RONS	Relative Gut Size vs. MTT	Relative Gut Size vs. GFP Intensity
Pearson r							
r	-0.7130	0.8050	0.3293	-0.9580	-0.7911	-0.8756	-0.8500
95% confidence interval	-0.9934 to 0.7882	-0.6896 to 0.9957	-0.9243 to 0.9802	-0.9991 to 0.03926	-0.9954 to 0.7093	-0.9974 to 0.5395	-0.9968 to 0.6068
R squared	0.5083	0.6481	0.1084	0.9177	0.6258	0.7666	0.7225
P value							
P (two-tailed)	0.29	0.19	0.67	0.04	0.21	0.12	0.15
Significant? (alpha = 0.05)	No	No	No	Yes	No	No	No

Table S7: Correlation analysis of gut size and assayed biomarkers of trametinib-treated *byn>RAP-N4* fly avatar.

	Relative Gut Size vs. ECLOSION	Relative Gut Size vs. TSH	Relative Gut Size vs. NPSH	Relative Gut Size vs. NO	Relative Gut Size vs. RONS	Relative Gut Size vs. MTT	Relative Gut Size vs. GFP Intensity
Pearson r							
r	-0.5563	0.6534	-0.8380	-0.5902	-0.9058	0.1335	-0.6918
95% confidence interval	-0.9887 to 0.8699	-0.8270 to 0.9917	-0.9965 to 0.6324	-0.9898 to 0.8570	-0.9980 to 0.4272	-0.9494 to 0.9701	-0.9928 to 0.8036
R squared	0.3094	0.4270	0.7023	0.3483	0.8204	0.01783	0.4786
P value							
P (two-tailed)	0.44	0.35	0.16	0.41	0.09	0.87	0.31
Significant? (alpha = 0.05)	No	No	No	No	No	No	No

Table S8: Correlation analysis of relative gut size and assayed biomarkers of regorafenib-treated *byn>RAP-N11* fly avatar

	Relative Gut Size vs. Eclosion	Relative Gut Size vs. TSH	Relative Gut Size vs. NPSH	Relative Gut Size vs. NO	Relative Gut Size vs. RONS	Relative Gut Size vs. MTT	Relative Gut Size vs. GFP Intensity
Pearson r							
r	-0.9326	0.8716	0.8378	-0.8423	0.1194	0.09112	-0.5165
95% confidence interval	-0.9986 to 0.2750	-0.5514 to 0.9973	-0.6329 to 0.9965	-0.9966 to 0.6237	-0.9508 to 0.9693	-0.9535 to 0.9675	-0.9874 to 0.8828
R squared	0.8697	0.7597	0.7019	0.7095	0.01427	0.008303	0.2668
P value							
P (two-tailed)	.067	.128	.162	.158	.881	.909	.483
Significant? (alpha = 0.05)	No	No	No	No	No	No	No

Table S9: Correlation analysis of gut size and assayed biomarkers of trametinib-treated *byn>RAP-N11* fly avatar.

	Relative Gut Sizes vs. Eclosion	Relative Gut Sizes vs. TSH	Relative Gut Sizes vs. NPSH	Relative Gut Sizes vs. NO	Relative Gut Sizes vs. RONS	Relative Gut Sizes vs. MTT	Relative Gut Sizes vs. GFP Intensity
Pearson r							
r	-0.9162	0.8182	0.6122	-0.7078	-0.2854	0.09342	-0.6259
95% confidence interval	-0.9983 to 0.3760	-0.6688 to 0.9960	-0.8476 to 0.9905	-0.9932 to 0.7922	-0.9782 to 0.9311	-0.9533 to 0.9676	-0.9909 to 0.8412
R squared	0.8393	0.6694	0.3747	0.5010	0.08148	0.008727	0.3918
P value							
P (two-tailed)	0.08	0.18	0.39	0.29	0.71	0.91	0.37
P-value summary	ns	ns	ns	ns	ns	ns	ns
Significant? (alpha = 0.05)	No	No	No	No	No	No	No
